# BLAST from the Past: Impacts of Evolving Approaches on Studies of Evolution by Gene Duplication

**DOI:** 10.1093/gbe/evab149

**Published:** 2021-06-23

**Authors:** Frédéric J J Chain, Raquel Assis

**Affiliations:** 1 Department of Biological Sciences, University of Massachusetts Lowell, Massachusetts, USA; 2 Department of Electrical Engineering and Computer Science, Florida Atlantic University, Boca Raton, Florida, USA; 3 Institute for Human Health and Disease Intervention, Florida Atlantic University, Boca Raton, Florida, USA

**Keywords:** duplicate genes, paralogs, gene families, copy number, evolutionary genomics

## Abstract

In 1970, Susumu Ohno hypothesized that gene duplication was a major reservoir of adaptive innovation. However, it was not until over two decades later that DNA sequencing studies uncovered the ubiquity of gene duplication across all domains of life, highlighting its global importance in the evolution of phenotypic complexity and species diversification. Today, it seems that there are no limits to the study of evolution by gene duplication, as it has rapidly coevolved with numerous experimental and computational advances in genomics. In this perspective, we examine word stem usage in PubMed abstracts to infer how evolving discoveries and technologies have shaped the landscape of studying evolution by gene duplication, leading to a more refined understanding of its role in the emergence of novel phenotypes.

## Introduction

Significance

Gene duplication is a frequent mutational process that plays a key role in the evolution of new biological functions. In this perspective, we examine word stem usage in PubMed-indexed articles to elucidate how evolving approaches shaped the landscape of studying evolution by gene duplication over time. Our analysis illustrates that experimental and computational advances in genomics widened the research scope, fueling transitions from single-gene sequence studies in a handful of species to genome-wide functional interrogations in numerous model and non-model systems, thereby enhancing our knowledge of the evolutionary outcomes of gene duplication across the tree of life.

**Fig. 1.**
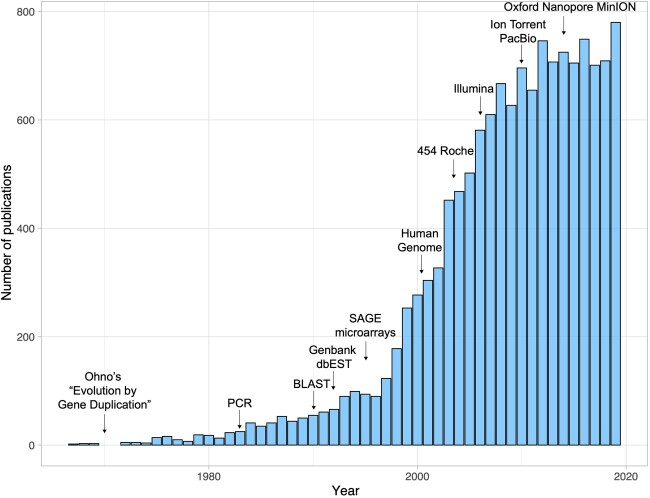
Number of publications in PubMed with terms related to both gene duplication and evolution in their titles or abstracts. A search was performed on July 1, 2020 using the query ((“gene duplic*” OR “duplicate gene*” OR paralog* OR “duplicated gene*” OR “gene cop*”) AND (evolution* OR evolv*)). The output was restricted to full calendar years, yielding a total of 13,919 articles published between 1967 and 2019. Major technological developments and innovations are indicated by arrows.

Gene duplication is often hailed as a key driver of evolution. Indeed, approximately 40% of all PubMed titles or abstracts with word stems related to gene duplication also reference evolution. Although numerous ideas and articles on evolution by gene duplication were published in the first half of the 20th century (reviewed by Taylor and Raes 2004), it was not until 1967 that gene duplication and evolution word stems appeared together in an article indexed by PubMed (fig. 1; Black and Dixon 1967). Even so, this article was succeeded by only a handful of others during the next 2 years. Then, in 1970, Susumu Ohno published his landmark book, “Evolution by gene duplication,” in which he hypothesized that gene duplication plays a major role in evolution (Ohno 1970). Specifically, he argued that gene duplication creates a redundant gene copy that is released from the hold of natural selection, allowing it to accumulate previously “forbidden mutations” that can lead to its acquisition of a new function (Ohno 1970). Ohno’s book fueled interest in evolution by gene duplication, creating a new research niche at the interface of evolutionary biology and genetics. Nevertheless, limited and time-consuming experimental approaches impeded progress in the field, with fewer than 20 articles published per year during the following decade.

It was not until Kary Mullis’ invention of the polymerase chain reaction (PCR) in 1984 that researchers were able to easily isolate and sequence genes (Mullis 1990), fueling a rapid increase in molecular studies of duplicate genes. The initial step in bioinformatic identification of duplicate genes from sequence data is homology detection, which is often followed by analyses of additional features, such as gene structure and synteny (Byrne and Wolfe 2005). Many sequence alignment tools existed for homology detection when PCR was introduced (e.g., Needleman and Wunsch 1970; Smith and Waterman 1981; Lipman and Pearson 1985) and were fundamental in early studies incorporating small numbers of DNA sequences. However, the growing abundance of DNA sequence data introduced a new obstacle to research on evolution by gene duplication—the lack of computational tools for rapidly quantifying genetic similarities among many sequences. Fortunately, this problem was solved with the development of the basic local alignment search tool (BLAST; Altschul et al. 1990), which enabled more efficient identification and comparison of duplicate gene sequences within and across species than the only previous approach for this task (Lipman and Pearson 1985). BLAST proved essential in initial large- and genome-scale studies of the molecular evolution of duplicate genes (Lynch and Conery 2000), and continues to be useful for evaluating orthologous and paralogous relationships. The following two decades saw an explosive growth in evolutionary studies of gene duplication, as PCR and the availability of BLAST and a host of other important sequencing alignment tools (e.g., Needleman and Wunsch 1970; Smith and Waterman 1981; Lipman and Pearson 1985; Thompson et al. 1994) set the stage for many exciting developments in genome sequencing and analysis.

Technological and computational advances opened up a wider research scope in the postgenomic era, prompting a transition from single gene to genome-wide studies. Though the rate of research on evolution by gene duplication appears to have stabilized in the last decade, the equilibrium number of publications per year represents a growth of over two orders of magnitude since its inception. More importantly, papers published today share little in common with their ancestors, as studies rapidly coevolved with experimental and computational advances in the field. Consequently, modern studies employ a much more sophisticated toolkit that permits researchers to address many previously intangible hypotheses about gene duplication in a diversity of biological systems, yielding an enhanced and nuanced understanding of evolution by gene duplication across the tree of life.

## Evolution of Research Scope

**Fig. 2.**
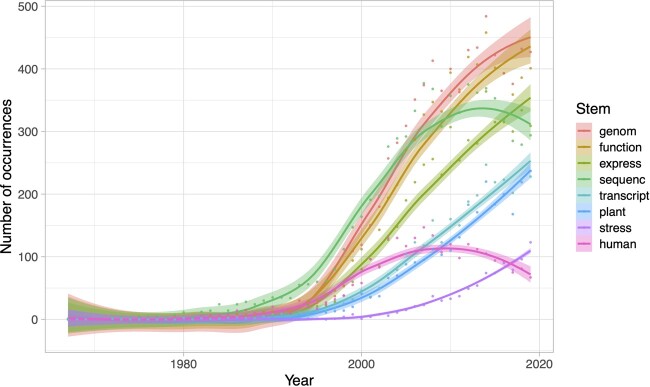
Number of occurrences of word stems with large proportional changes over time in titles and abstracts referencing gene duplication and evolution. Stems of all words in titles and abstracts used in figure 1 were extracted, and their frequencies were computed for each year between 1967 and 2019. Stems are ordered based on their frequencies in 2019.

The first PubMed-indexed article to reference both gene duplication and evolution in its title or abstract describes a metabolic protein in the Pacific Herring (*Clupea pallasi*) with homologous regions hypothesized to have arisen by four successive partial gene duplication events (Black and Dixon 1967). Other initial publications are similarly limited in scope, in that they primarily compose case studies of individual genes and gene families in single species. In contrast, recent publications often describe genome-scale comparisons across multiple related species. This transition has been enabled by technological advancements in both generating and analyzing next-generation sequencing data, which together have provided a foundation for testing and refining important theoretical models of duplicate gene evolution, such as neofunctionalization (Ohno 1970), subfunctionalization (Force et al. 1999; Stoltzfus 1999), and dosage balance (Birchler et al. 2005). To take a closer glimpse into how the research scope has evolved during the past half century, we investigated word stem usage over time in PubMed-indexed titles and abstracts of articles about evolution by gene duplication (fig. 2). Examinations of eight focal word stems with large proportional changes in frequencies over this time interval reveal three interesting trends.

First, changes in the frequencies of stems related to DNA sequencing correspond well with advancements in sequencing technologies and associated computational tools. The frequency of the stem “sequenc” began increasing around the time of the introduction of PCR in 1984, and more rapidly around the time of the development of BLAST in 1990 (Altschul et al. 1990), at which point it was included in approximately 83% of all articles in PubMed. It continued increasing at a slower pace around the time of the release of the Roche 454 sequencing technology in 2005, stabilized for several years while new short-read (e.g., Illumina in 2006) and long-read (e.g., PacBio in 2011 and MinION in 2014) sequencing methods were introduced, and then began decreasing during the last few years. Though “sequenc” is still present in the main texts of many published articles, the sheer abundance of sequence data today likely precludes the need to mention it in titles or abstracts. In contrast, the frequency of the stem “genom” began increasing a few years after “sequenc,” though at a faster rate, and continued increasing while “sequenc” decreased. It is also the most common stem in titles and abstracts of publications on evolution by gene duplication in the past decade. The observed divergence between the frequencies of these two stems can likely be attributed to the gradual replacement of small-scale with genome-scale analyses over time. In particular, whereas PCR and BLAST facilitated evolutionary comparisons of duplicate gene sequences during the early 1990s, advancements in sequencing technologies enabled these same analyses to be performed on all duplicate genes across genomes beginning in the mid to late 1990s. For example, the pioneer of these studies showed that almost half of *Escherichia coli* proteins likely arose through gene duplication (Labedan and Riley 1995), representing our first glimpse into the global importance of gene duplication. Over time, the movement toward genome-scale studies accelerated as new sequencing technologies and tools emerged and became more affordable. By offering a global perspective, genome-scale studies led to many fundamental discoveries about the evolution of duplicate genes, including their diverse mutational mechanisms (Zhang 2003; Katju 2012), rapid mutation rates (Lynch et al. 2008; Katju and Bergthorsson 2013; Schrider et al. 2013) and evolutionary rates (Kondrashov et al. 2002; Conant and Wagner 2003; Chain and Evans 2006), and ubiquity in taxa from all domains of life (Kondrashov et al. 2002; Zhang 2003; Kondrashov 2012). The postgenomic era has helped confirm the importance of gene duplication across a multitude of biological systems, improving our appreciation for the various ways in which it impacts evolutionary innovation.

Second, changes in word stem frequencies reflect a recent switch in the focus of studies from sequence to function. Specifically, the stems “function,” “express,” and “transcript” display the greatest changes in frequencies over time along with “genom” and “sequenc.” As with “genom,” the frequency of each of these stems began rapidly increasing in the mid-1990s and continued increasing until today. The simultaneous increases in frequencies of all three of these stems along with “genom” exemplifies the integration of functional genomics, particularly transcriptomics and gene expression quantification, in many contemporary studies of evolution by gene duplication. This shift in approach comprises a major turning point in the field, in that it has given researchers a unique opportunity to address Ohno’s hypothesis that gene duplication is a major source of new gene functions (Ohno 1970). Whereas earlier sequence-based studies identified rapid and asymmetric genetic divergence consistent with this hypothesis (Kondrashov et al. 2002; Conant and Wagner 2003; Chain and Evans 2006), expression data provide a more direct source of functional information about genes because they allow us to measure their activity levels across space and time. Early work in this area compared expression profiles between duplicate gene copies, finding that many are substantially different from one another in level or spatial distribution (Gu et al. 2002; Li et al. 2005; Chain et al. 2008). Later studies employing new phylogenetic approaches (Assis and Bachtrog 2013; DeGiorgio and Assis 2021) showed that rapid and asymmetric multitissue expression divergence is a common outcome of gene duplication (Assis and Bachtrog 2013, 2015; Chau and Goodisman 2017; Jiang and Assis 2019; DeGiorgio and Assis 2021). Further, experimental modification of gene regulation with modern functional genomics approaches, such as RNAi and CRISPR, have directly linked new duplicate gene functions to phenotypes (Chen et al. 2010, 2019; Turetzek et al. 2016; Naseeb et al. 2017). Together, these studies support Ohno’s hypothesis that gene duplication is a major source of new gene functions and phenotypes.

Third, changes in word stem frequencies point to a dramatic shift in popular study systems. In particular, two word stems are related to study systems—“plant” and “human.” Though the frequency of human initially increased at a faster rate, it reached its peak frequency in 2007 (*n *=* *147) and steadily decreased afterward. In contrast, plant continued increasing and surpassed human in 2011 (*n *=* *131 vs. *n *=* *98), reaching 237 occurrences by 2019. We do not believe that the concurrent rise of plant and fall of human is attributed to greater biological interest or availability of genomic data in plants than in humans. Rather, we hypothesize that this transition was propelled by the confirmation from next-generation sequencing studies that plants underwent several rounds of whole-genome duplication, including recent events that may have contributed to domestication for agriculture (Glémin and Bataillon 2009; Panchy et al. 2016). Though polyploidy is also common in many other taxa (Simakov et al. 2020), its predominance across plant species provide numerous useful study systems for research on homolog divergence, genetic and phenotypic impacts of evolution by gene duplication, and differences in retention mechanisms and rates of duplicate genes derived from small-scale versus whole-genome duplication events (e.g., Maere et al. 2005). Further, the frequency of the stem “stress” also increased during the past decade. Of articles with the stem stress, 58% (507/881) also contain the stem plant ($\chi^{2}$ test, p<0.001). This is not surprising, as stress response to changing environmental conditions and pathogens is an important area of research in plants, particularly in efforts to enhance agricultural productivity and sustainability (Zhu 2016). Additionally, 79% (692/881) of articles with the stem stress contain either the stem “express” or “transcript” ($\chi^{2}$test, p<0.001). In combination, these cooccurrences highlight the application of interdisciplinary approaches in addressing how gene duplication contributes to adaptive stress response in plants using the growing availability of genomic data and tools.

## Evolution of Study Systems

**Fig. 3.**
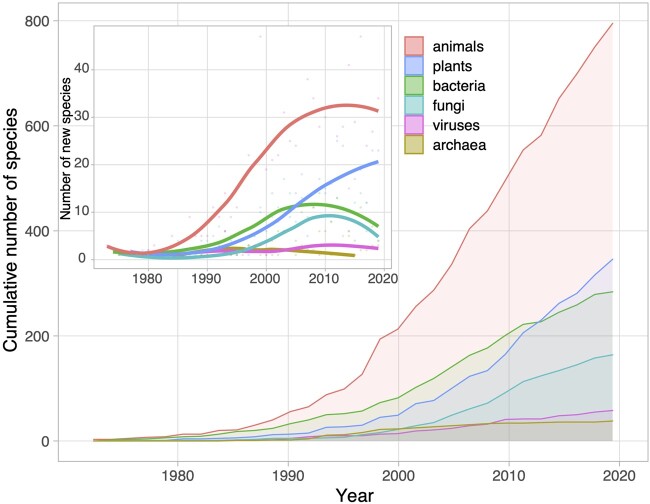
Numbers of total species and new species from six taxonomic groups in titles and abstracts referencing gene duplication and evolution. Linnaean names of species from titles and abstracts used in figure 1 were extracted with Linnaeus v 2.0 (Gerner et al. 2010), and full taxonomic lineages with taxize (Chamberlain and Szöcs 2013). Species were binned into six taxonomic groups based on their frequencies: animals, plants, bacteria, fungi, viruses, and archaea. The main plot shows the cumulative number of species, and the inset plot shows the number of new species, from each taxonomic group over time.

Temporal transitions in the scope of studies on evolution by gene duplication from single gene to whole genome, and then from genome to transcriptome, are consistent with the technological advances made over the years. However, the observed negative correlation between frequencies of analyses in humans and plants during the postgenomic era could be a symptom of more intricate changes in study systems. Did the rise of sequencing technologies and recent emergence of functional genomic techniques lead to a broadening in the diversity of study systems that are interrogated in examinations of evolution by gene duplication? To address this question, we scoured titles and abstracts of articles referencing gene duplication and evolution for changes in relative contributions of species from six taxonomic groups: animals, plants, bacteria, fungi, viruses, and archaea (fig. 3).

Our analysis reveals that, whereas animals continue to be the most studied taxonomic group, new plant species have been added to the literature on evolution by gene duplication at consistently high rates during the past decade. Another important observation is that the total number of new species in the literature has increased substantially since 1967. Until 1990, there was approximately one new species mentioned per four publications, whereas the next decade saw almost one new species every two publications. Since 2000, there has been a steady contribution of one new species every three publications, likely reflecting the availability of cheaper and more efficient whole-genome sequencing technologies that allow researchers to tackle research problems in previously uninterrogated organisms. The resulting diversity of study systems enriches our understanding of evolution by gene duplication across species and taxonomic boundaries, such as how multiple whole-genome duplication events during the last 500 Myr have shaped contemporary vertebrate genome structure (Simakov et al. 2020).

Moreover, having a broader array of study systems promotes the investigation of complex questions that may not be accessible in most animal and plant systems. For example, an assortment of organisms have been used to test evolutionary models of duplicate genes under different ecological contexts in lineages inhabiting different natural environments (Miller et al. 2011; Eberlein et al. 2017) or experimentally induced environmental gradients (Chow et al. 2012; Toll-Riera et al. 2016). Additionally, long-term laboratory experiments in several species have uncovered high basal rates of gene duplication (Lynch et al. 2008; Schrider et al. 2013; Katju and Bergthorsson 2013) and fitness tradeoffs of whole-genome duplications (Fisher et al. 2018). It is also clear that the integration of data from diverse taxonomic groups has transformed studies on the evolution of duplicate genes by shedding light on genomic phenomena, such as the role of DNA methylation in maintaining young duplicate genes (Chang and Liao 2012; Keller and Yi 2014; Huang and Chain 2021), and by unifying key evolutionary concepts, such as the evolution of multicellularity (Oud et al. 2013; Stiller et al. 2018) and evolution of sex (Connallon and Clark 2011; Rafati et al. 2020).

## Future Trajectory of Research

Given the dynamic research landscape witnessed to date, what will fuel another explosion of interest in the study of evolution by gene duplication analogous to that experienced in the mid-1990s? Though BLAST remains useful for inferring orthologous and paralogous relationships and continues to be a prominent tool embedded in genomic databases, the recent rise of interdisciplinary experimental and computational approaches is broadening our ability to assay functional impacts of duplicate genes. Thus, we hypothesize that transformative discoveries about evolution by gene duplication will be made via the introduction and exploitation of novel technologies that enable us to interrogate duplicate gene functions through examinations of expression divergence at single-cell resolution (e.g., Coate et al. 2020) and systems-level impacts of their copy number variations (CNVs) segregating within populations (e.g., Huang et al. 2019). In particular, recent developments in genetic modifications allow for a deeper understanding of the mechanisms and functional consequences of CNVs, maneuvering the field toward experimental manipulations of genomes to study new duplications in both model and nonmodel organisms. The availability of long-read sequencing approaches (e.g., Pacific Biosciences “single-molecule real-time sequencing” and Oxford Nanopore Technologies “nanopore sequencing”) and long-range scaffolding technologies (e.g., 10X linked reads, Hi-C, and Bionano optical mapping) further permit phasing and resolving the structural composition of duplications along chromosomes, increasing the resolution capabilities for characterizing CNVs with little to no sequence divergence (Hoff et al. 2019). This combination of evolving technologies and taxonomic diversity in which researchers can carry out experimental and computational studies promises an exciting future for discoveries about the evolutionary and phenotypic impacts of gene duplication.
